# Responses of Aquatic Bacteria to Terrestrial Runoff: Effects on Community Structure and Key Taxonomic Groups

**DOI:** 10.3389/fmicb.2016.00889

**Published:** 2016-06-15

**Authors:** Huong T. Le, Cuong T. Ho, Quan H. Trinh, Duc A. Trinh, Minh T. N. Luu, Hai S. Tran, Didier Orange, Jean L. Janeau, Asmaa Merroune, Emma Rochelle-Newall, Thomas Pommier

**Affiliations:** ^1^Ecologie Microbienne, INRA, UMR1418, CNRS, UMR5557, Université Lyon 1Villeurbanne, France; ^2^Department of Hydrobiology, Institute of Environmental Technology, Vietnam Academy of Science and TechnologyHanoi, Vietnam; ^3^iEES-Paris (IRD, Sorbonne Universités, UPMC Univ Paris 06, CNRS, INRA, UPEC, Université Paris Diderot)Paris, France; ^4^Laboratory of Analytical Science, Institute of Chemistry, Vietnam Academy of Science and TechnologyHanoi, Vietnam; ^5^Soils and Fertilizers Research InstituteHanoi, Vietnam; ^6^IRD, UMR 242, Institute of Ecology and Environmental Sciences – Paris, c/o Soils and Fertilizers Research InstituteHanoi, Vietnam; ^7^IRD, UMR 210 Eco&SolsMontpellier, France

**Keywords:** DOC, compost, biochar, aquatic microbial community, mesocosms

## Abstract

Organic fertilizer application is often touted as an economical and effective method to increase soil fertility. However, this amendment may increase dissolved organic carbon (DOC) runoff into downstream aquatic ecosystems and may consequently alter aquatic microbial community. We focused on understanding the effects of DOC runoff from soils amended with compost, vermicompost, or biochar on the aquatic microbial community of a tropical reservoir. Runoff collected from a series of rainfall simulations on soils amended with different organic fertilizers was incubated for 16 days in a series of 200 L mesocosms filled with water from a downstream reservoir. We applied 454 high throughput pyrosequencing for bacterial 16S rRNA genes to analyze microbial communities. After 16 days of incubation, the richness and evenness of the microbial communities present decreased in the mesocosms amended with any organic fertilizers, except for the evenness in the mesocosms amended with compost runoff. In contrast, they increased in the reservoir water control and soil-only amended mesocosms. Community structure was mainly affected by pH and DOC concentration. Compared to the autochthonous organic carbon produced during primary production, the addition of allochthonous DOC from these organic amendments seemed to exert a stronger effect on the communities over the period of incubation. While the *Proteobacteria* and *Actinobacteria* classes were positively associated with higher DOC concentration, the number of sequences representing key bacterial groups differed between mesocosms particularly between the biochar runoff addition and the compost or vermi-compost runoff additions. The genera of *Propionibacterium* spp. and *Methylobacterium* spp. were highly abundant in the compost runoff additions suggesting that they may represent sentinel species of complex organic carbon inputs. Overall, this work further underlines the importance of studying the off-site impacts of organic fertilizers as their impact on downstream aquatic systems is not negligible.

## Introduction

Organic fertilizers are cited as being a sustainable option for improving soil quality and crop yields in degraded soils (Eghball, 2002). Many studies have shown that the incorporation of compost or vermi-compost into soil improves water retention, cation exchange capacity, soil structure, organic carbon content, and nutrient quality (Rivero et al., 2004). Compost and vermi-compost are formed by bio-oxidation and stabilization of vegetable and animal waste by microorganisms in the case of compost and through the joint action of earthworms and microorganisms in the case of vermi-compost (Vivas et al., 2009). Biochar, which is formed by the pyrolysis of organic matter such as bamboo or rice straw, is also used as a soil amendment and it is particularly promoted as a low-cost option in tropical countries. As with the other organic amendments, biochar has been shown to increase soil moisture retention, improve soil structure, and reduce nutrient leaching (Anderson et al., 2011; Doan et al., 2015) as well as reducing greenhouse gas emissions from soils (Lehmann, 2007).

Although the positive impacts of organic amendments on soil quality and crop yield in both temperate and tropical countries have been amply demonstrated, the impact of these amendments on soil runoff and the impact of that runoff on adjacent aquatic systems has been less studied. The concentration of dissolved organic carbon (DOC) in soil runoff is strongly influenced by soil organic carbon content, soil erosion, vegetation cover, amounts, and intensities of rainfall, as well as type of composting applied (Mailapalli et al., 2012; Worrall et al., 2012; Janeau et al., 2014; Pommier et al., 2014). It is therefore important to understand the fate of this DOC, as runoff has the potential to impact adjacent terrestrial and/or aquatic ecosystems (Jacinthe et al., 2004). Moreover, the impacts of agricultural runoff, e.g., nitrogen fertilizers, on adjacent aquatic systems have been the focus of many studies in temperate and developed countries for many years much less is known about the impacts of agricultural practices in the tropics. In addition, although many studies have examined the impacts of inorganic fertilizers (Howarth, 1998; Howarth et al., 2012) or pesticides and herbicides (Wohlfahrt et al., 2010; Dalton et al., 2015), less have focused on the impacts of organic fertilizers on downstream aquatic ecosystems. This is despite the known importance of organic matter for microbial processes, aquatic biogeochemistry and microbial community structure.

The changes in microbial composition and metabolic activity are probably tightly linked to shifts in the quantity and quality of DOM (Fang et al., 2014). Although the influence of changing dissolved organic matter (DOM) on bacterial diversity has been investigated in cultures (e.g., Sarmento et al., 2013), microcosms (Pommier et al., 2014) and coastal and oceanic waters (e.g., Cottrell and Kirchman, 2000; Gómez-Consarnau et al., 2012), a comprehensive understanding of how aquatic microbial communities respond to shifts in DOC bioavailability in runoff from agricultural soils in tropical environments is, to our knowledge, still lacking. Therefore, in this study, we investigated the impact of runoff from compost, vermi-compost and biochar on reservoir bacterial communities in mesocosm experiment. The objectives of this study were to determine how runoff from compost, vermi-compost and biochar influences aquatic bacterial communities in a typical tropical headwater reservoir. We hypothesized (i) that the application of organic fertilizer (compost, vermi-compost, and biochar) would lead to an increase in the concentration of DOC in runoff from agricultural soils and into the adjacent aquatic systems; (ii) that the addition of allochthonous DOC into a downstream water body would alter bacterial community structure, inducing the growth of key taxonomic groups that were adapted to different sources of DOC; and (iii) that biochar runoff, considering its different chemical structure, would induce different bacterial groups compared to compost and vermi-compost runoff additions.

## Materials and Methods

### Study Area

The rain simulation experiments were carried out in the peri-urban Dong Cao catchment, located in hills of Tien Xuan in the Thach That district of Ha Noi, Vietnam (20° 57′ 30″ N, 105° 29′ 15″ E; elevation 300 m a.s.l). The catchment has an area of 50 ha and is bisected by a small stream which is connected to a downstream reservoir (volume of 700,000 m^3^).

The dominant soil type in this catchment is an Acrisol, i.e., tropical clay-rich Ultisol (World Reference Base, 1998; Soil Survey Staff, 1999). The climate is a monsoon climate with the main precipitation falling within the summer from April to October. The 12 year (1999–2011) mean annual precipitation measured in this sub-catchment was 1502 mm year^-1^ with a minimum of 1262 mm year^-1^ in 2010 and a maximum of 2506 mm year^-1^ in 2001 (Podwojewski et al., 2008; Tran et al., 2011).

### Rainfall Simulations

The rainfall simulation took place in April 2012 at the end of the dry season. We examined in triplicate runoff and organic matter export under three types of amendment plus a soil only control (**Table 1**). The different amendments correspond to three different options that are promoted as improving soil organic carbon content and fertility: compost, vermi-compost and biochar.

**Table 1:** The different treatments applied to the plots and the average DOC concentration (mean + SE) in runoff from that treatment.

Practice	Addition	DOC (mg L^-1^ C)	TN (mg L^-1^ N)	TP (mg L^-1^ P)
Soil only	No addition	2.1 (0.3)^c^	1.6 (1.0)^a^	0.05 (0.02)^a^
+Compost	2 kg of compost	8.1 (2.3)^a^	1.5 (0.5)^a^	0.7 (0.6)^b^
+Vermicompost	2 kg of vermicompost	3.8 (0.9)^b,d^	1.8 (0.9)^a^	1.4 (0.3)^b^
+Biochar	340 g of biochar	2.6 (0.4)^c,d^	1.2 (0.5)^a^	0.08 (0.05)^a^

*Different letters indicate a significant difference in DOC, TN, or TP concentration. Each treatment was tested in triplicate.*

Rain simulations were conducted as in Janeau et al. (2014). Runoff water from the soil surface was drained from the bottom side of the plot via a drainage channel and was collected through a hole. Each plot was embedded approximately 10 cm into the soil surface and each frame was placed at between 4 and 6 m distances from the adjacent plots to ensure that there was no secondary influence from the other rain simulations. The plots were set out along a contour line in a field that is regularly used for crop cultivation. The slope of the plots varied between 28 and 45% with an average slope of 36.1 ± 8.5%.

For each simulation, two successive rain events were applied at a 24 h interval with a rain intensity of 90 mm h^-1^ during 40 min (∼585 J m^-2^). This intensity corresponds to an intense monsoonal rain event. Each plot was covered with PVC sheeting to avoid the influence of natural rainfalls occurring between the simulations.

Runoff water from the 1 m^2^ plot was collected in a large, clean bucket during each rain event. At the end of each of the two simulations per plot, 5 L of runoff water was collected and stored at 4°C for use in the aquatic mesocosm incubation (see below). Total nitrogen (TN), total phosphorus (TP), and DOC concentrations were measured at the end of each rain simulation.

### Aquatic Mesocosm Incubation

In order to assess the impact of runoff water from different amendments on aquatic microbial communities, a short, 16 days mesocosm incubation was conducted immediately after the rain simulations. Fifteen plastic containers of 200 L volume corresponding to the 3 replicates of the 3 organic addition treatments plus the 3 soil only controls and 3 replicate reservoir water controls were filled with 100 L of water collected from the reservoir in the studied catchment.

Ten liters of runoff, 5 L from each of the two simulations, was added at the beginning of the incubation to the respective mesocosms, except for the reservoir controls. These additions were calculated based on the data of Bui et al. (2014) on runoff of water from the watershed to the reservoir and on annual precipitation volumes. Annual rainfalls in 2012 and 2013 in the Dong Cao watershed accounted for 1,200 and 1,800 mm, respectively, of which between 80 and 90% occurred over the 5-month period between May and September. While the mean peak flow of water runoff to the lake is estimated around 0.11 m^3^ s^-1^ (i.e., 10,000 m^3^ day^-1^; Bui et al., 2014), the volume of the reservoir was estimated to seasonally change between 640,000 and 1,120,000 m^3^ (Trinh et al., 2016). In other words, over the 5-month rainy season the water volume increases by approximately 43% (480,000 m^3^), the majority of which comes from runoff. Compared to natural conditions, the chosen 10 L-addition of runoff water to 100 L-reservoir water therefore represents a typical addition occurring during one month of the rainy season.

Each mesocosm was covered with one layer of mosquito screening to protect from falling debris such as leaves and the incubation was conducted outside and ran for 16 days. Mesocosms were mixed manually daily and samples for nutrients, DOC, and Chlorophyll *a* (Chl*a*) concentration (days 0, 1, 2, 3, 5, 7, and 16) and bacterial diversity (*T*_i_ = Day 0 and *T*_f_ = Day 16) were collected. Temperature, dissolved oxygen (DO) and total dissolved solids (TDS) were measured daily with a Hydrolab 5 multiprobe in each mesocosm after mixing.

### Nutrients and DOC

For the determination of DOC concentration, duplicate 30 mL samples were filtered (Whatman GF/F), collected in pre-combusted (450°C, overnight) glass tubes, preserved with 36 μL 85% phosphoric acid (H_3_PO_4_) and sealed with a Teflon-lined cap. Samples were stored at ambient temperature and in the dark until measurement. DOC concentration was measured on a Shimadzu TOC *V*_CPH_ analyzer following the method described in Rochelle-Newall et al. (2011).

Total nitrogen and TP concentrations were determined following the standard methods for the examination of water and wastewater (Clesceri et al., 1999). TN was measured on unfiltered samples using the Total Kjeldahl Nitrogen (TKN) method (4500-Norg B) (Clesceri et al., 1999). TP was determined on unfiltered samples after addition of concentrated HClO_4_ solution and heating at 150°C for 2 h followed by determination of PO_4_ colorimetrically at 880 nm with the ascorbic acid method (Standard method 4500-P E, Clesceri et al., 1999). All colorimetric measurements were conducted on a GBC Cintra 40 spectrophotometer (Australia). Concentrations of Chl*a* were determined fluorometrically from 30 mL subsamples filtered onto 25 mm Whatman GF/F filters according to Holm-Hansen et al. (1965). The rainwater used for the simulations was taken from a nearby spring and the concentrations of DOC (0.74 mg C L^-1^), TN (0.024 mg L^-1^) and TP (0.025 mg L^-1^) were subtracted from the DOC, TN, and TP runoff measurements.

### DNA Extraction and 16S rRNA Gene Sequencing

Samples of 50 mL from each incubation were filtered onto separate 0.2 μm (ø = 47 mm) Whatman Nuclepore polycarbonate membranes at the initial (*T*_i_) and final time (*T*_f_) of the incubation. DNA was extracted using an in-house standard protocol. Filters were thawed and lysozyme (1 mg mL^-1^ final) was added and left for 30 min at 37°C. Sodium dodecyl sulfate (SDS, 1% final) and proteinase K (100 μg mL^-1^ final) were added and incubated overnight at 55°C. Cell lysates were extracted with an equal volume of phenol/chloroform/isoamyl alcohol [25:24:1] mixed thoroughly and centrifuged for 5 min (20,000 g). The upper phase of the centrifugation products were transferred to new tubes and an equal volume of chloroform/isoamylalcohol [24:1] was added and centrifuged for 5 min (20,000 g). The upper phase of the centrifugation products were transferred to new tubes and roughly 1/10 volume equivalent of sodium acetate (NaAc) [3M, pH = 5.2] was added together with 0.6 volume equivalent of isopropanol and incubated 1 h at –20°C. A new centrifugation was performed at 20,000 g for 20 min at 4°C. The pellet was washed with ice cold 70% ethanol, dried in a SpeedVac concentrator (Savant), re-suspended in 50 μL TE buffer, and quantified using the PicoGreen^®^ fluometric quantification kit (molecular Probes). PCR amplification of the hypervariable regions V1–V3 of the 16S rRNA gene was performed using the primers 27F (5′-GAGTTTGATCMTGGCTCAG-3′) and 518R (5′-WTTACCGCGGCTGCTGG-3′). Prior to pyrosequencing all PCR amplicons were pooled to equimolar ratio. The forward and reverse primers included both a 10 bp multiplex identifier (MID) in order to multiplex the samples during sequencing. Amplifications were performed in triplicate using the AmpliTaq Gold^®^360 master mix (Applied Biosystems), according to the protocol of the manufacturer. Cycling conditions were as follows: an initial activation/denaturation step at 95°C for 10 min; followed by 25 cycles of 95°C for 40 s, 55°C for 40 s, and 72°C for 1 min; and a final 7-min extension at 72°C. PCR products were then purified using the QIAquick PCR Purification Kit (Qiagen, Hilden, Germany) after excision of the amplicon from an agarose gel. Concentration of DNA of each identified PCR product was determined using Picogreen quantification and then PCR triplicates of a same site were pooled into equimolar concentrations. Pyrosequencing was then performed on a 454 GS-FLX Titanium (454 Life Sciences) by Macrogen, Korea. Data are publicly available at DDBJ/ENA/GenBank under the accession KAFM01000001-KAFM01012423.

### Sequences Processing and Data Analysis

The MOTHUR software (v. 1.33; Schloss et al., 2009) was used to process 16S rRNA gene sequence reads. Short reads (<250 bp) and reads with ambiguous primer or barcode sequences were discarded. Sequencing errors were reduced by aligning remaining reads to the SILVA database (Pruesse et al., 2007), screening the alignment to the overlapping region, and pre-clustering sequences distant by <2 bp. Chimeric sequences were identified using the integrated version of UChime (Edgar et al., 2011) and removed accordingly. To avoid misinterpretation, sequences that were classified as “Chloroplast”, “Mitochondria”, or “unknown” lineages were removed before clustering into Operational taxonomic units (OTUs). Sequences with a pairwise distance <0.03 substitutions per nucleotide were clustered in OTU and considered for further analyses. The estimated richness and evenness were calculated through Chao and invSimpson indices, respectively. Taxonomic assignments were performed on the alignment of consensus sequences with the RDP database (Cole et al., 2005).

To assess the relative influence of environmental parameters and changes in community structure, a canonical correspondence analysis (CCA) was performed between key environmental parameters (pH, TDS, DO, and DOC) and community composition using R with the ‘vegan’ package (Dixon, 2003). A heatmap was performed to show the comparison and cluster analysis of microbial composition in the pool samples at the genus level using R with the “pheatmap” package (Kolde, 2012).

### Statistical Tests

Significant differences between the treatments were tested using the Xlstat 2012 (Addinsoft) software. Analysis of variance (ANOVA) was used to test the significance of the differences between treatments after checking that the assumptions of the ANOVA were met. When necessary, the data were log-transformed to assure normality. When a significant difference was observed, an *a posteriori* test (Fisher’s LSD) was used to distinguish groups. When transformation did not assure normality, a non-parametric Kruskal–Wallis test was used.

Metastats was used to test the difference of abundance to identify significantly abundant genera in the samples. Analysis of Similarity (ANOSIM) was used to test the effect of time on community structure in all samples.

## Results

### DOC in Runoff

There were significant differences in the amount of DOC lost as runoff during the rain simulation between amendments (*p* < 0.05; **Table 1**). Compared to the soil only treatment (2.1 + 0.3 mg L^-1^ C), DOC was over three times higher in the runoff from plots with compost (8.1 + 2.3 mg L^-1^ C). TN and TP also varied between treatments with the concentrations being lowest in the soil only runoff for TN and in the soil only and biochar runoff for TP.

### Environmental Variables of Aquatic Mesocosms

**FIGURE 1:**
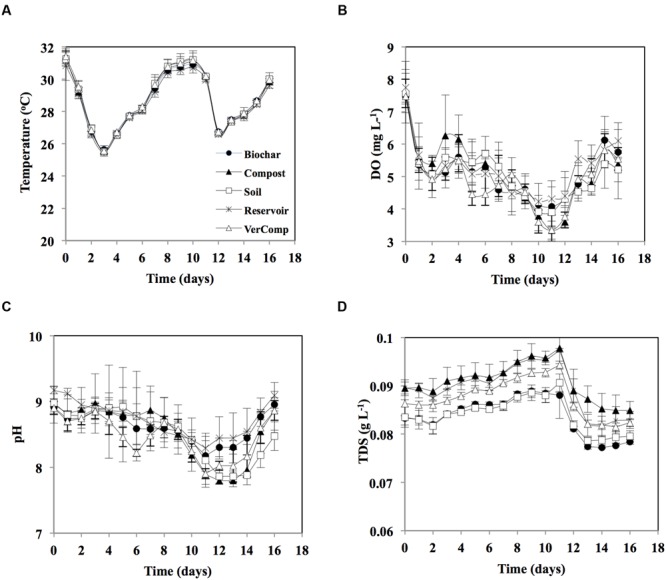
**Environmental parameters during 16 days incubation. (A)** Temperature (°C); **(B)** Dissolved oxygen (DO; mg L^-1^); **(C)** pH; and **(D)** Total dissolved solids (TDS, mg L^-1^). The mean and standard deviation is given for each treatment (*n* = 3).

The temporal variation of the measured environmental parameters (temperature, DO, pH, and TDS) was rather similar amongst treatments (**Figure 1**). Average temperature varied between 26 and 31°C over the course of the experiment (**Figure 1A**). DO concentration decreased from around 7.5 at day 0 to about 4 at day 12, thereafter it slightly increased to reach 5.5 by the end of the experiment (**Figure 1B**). pH varied between 7.8 and about 9 in all samples, decreasing from around 9 at day 0 to around 8 by days 11–13, it increased again to almost 9 on the last day of incubation (**Figure 1C**). TDS in the compost treatment was slightly higher than other treatments in all days of incubation. The TDS concentration gradually rose and was highest on day 11 (0.09 g L^-1^), thereafter it decreased to 0.07 g L^-1^ by the end of the experiment (**Figure 1D**).

### DOC and Chl*a*

**FIGURE 2:**
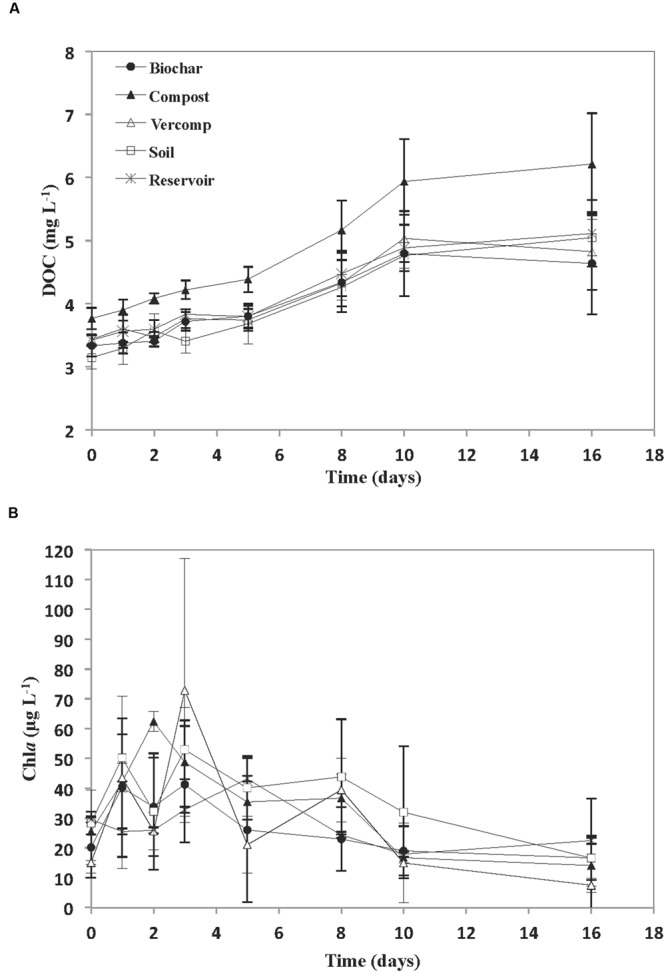
**(A)** Concentration of DOC (mg L^-1^) and **(B)** Chl*a* (μg L^-1^) for each treatment. The mean and standard deviation is given for each treatment (*n* = 3).

The addition of runoff to the reservoir water resulted in an increase in initial DOC concentration, relative to the reservoir (**Figure 2A**). This was particularly noticeable in the compost addition where initial concentrations of 3.7 + 0.3 mg C L^-1^ were observed. This was in contrast to an initial reservoir DOC concentration of 3.4 + 0.1 mg C L^-1^. The other additions resulted in initial DOC concentrations that varied between 3.1 + 0.2 mg C L^-1^ for the soil only runoff experiment and 3.4 + 0.01 mg C L^-1^ for the vermi-compost runoff addition. DOC concentration thereafter increased in all of the incubations. However, the rate of increase in DOC concentration was significantly higher in the compost addition (*p* < 0.01). Chl*a* was highly variable during the experiment with no significant differences between treatments (**Figure 2B**). In general, Chl*a* increased during the first few days to each a maximum on days 2–3 (46.2 + 15 μg L^-1^) in the runoff addition mesocosms and day 5 (43.2 + 21 μg L^-1^) in the reservoir water, and thereafter decreased towards the end of the experiment in all treatments (13.7 + 6 μg L^-1^).

### Estimated Richness, Evenness and Structure of Bacterial Community

**FIGURE 3:**
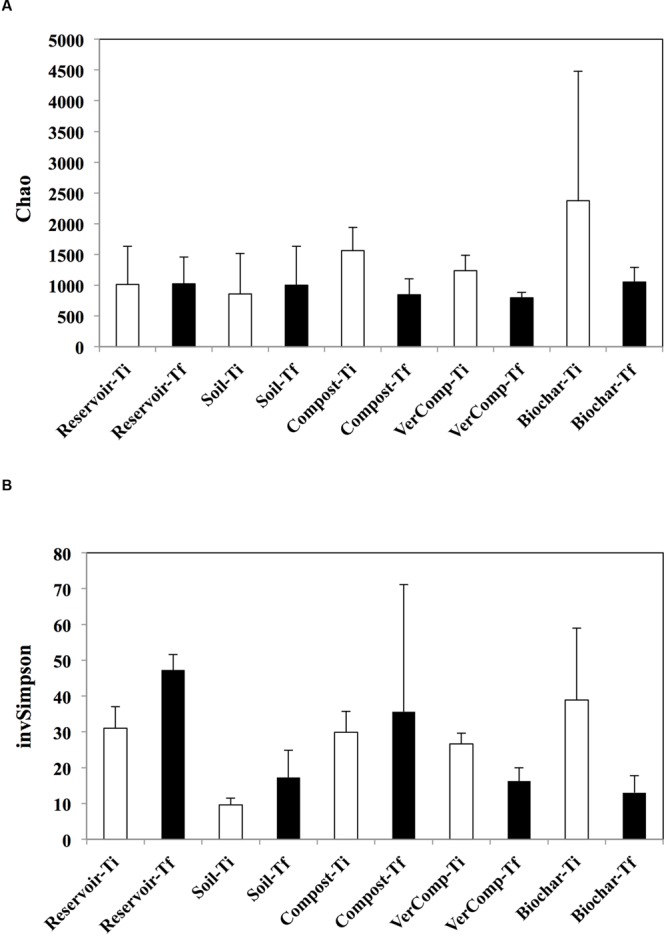
**Estimated richness and evenness. (A)** Chao index and **(B)** InvSimpson at *T*_i_ and *T*_f_ for all treatments. The mean and standard deviation is given for each treatment (*n* = 3).

In total, 327,327 sequences were obtained for all samples. At *T*_i_, the estimated richness was highest in the mesocosms receiving runoff water from soil fertilized with biochar. In these mesocosms, the richness strongly declined by 55.7% while it decreased by 46% in the compost addition and by 35.6 % in the vermi-compost addition between the two measured time points (**Figure 3A**). In contrast, richness did not vary in the control mesocosms that had received runoff water from soil only or those that were only filled with reservoir water. Correspondingly, evenness was negatively impacted in the mesocosms with biochar and vermicompost runoff additions between *T*_i_ and *T*_f_. However, evenness increased by 44% in the control mesocosms that had received runoffs from soil only and by 34% in the reservoir water controls. It also slightly increased in the mesocosms receiving runoff water from soil amended with compost by 15% but it is highly variable between these mesocosms (**Figure 3B**).

**FIGURE 4:**
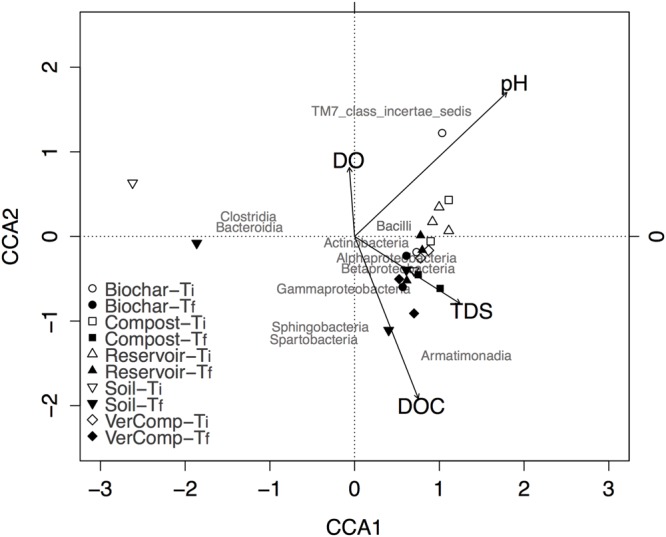
**Biplot of canonical correspondence analysis of taxa abundance with environmental parameters in all samples at class level.** Class of maximum abundance >1% at least in one sample was included in the analysis.

Also, community structure significantly changed through time (*P* < 0.01). The canonical correspondence analysis (CCA) between key environmental parameters (pH, TDS, DO, and DOC) and community composition showed that the proportion of the total variance explained by all environmental factors was 22.76%. The variance explained by the first three constrained axes (CCA1, CCA2, and CCA3) were 18%, 7.6% and 3.3%, respectively (**Figure 4**). CCA constructed at the taxonomic level of class indicated specific effects of environmental parameters on microbial community structure: DOC and pH were two important factors affecting microbial community (**Figure 4**). DOC was not significantly correlated with DO (*r* = 0.23, *P* = 0.2759), but was negatively correlated with TDS (*r* = –0.4, *P* = 0.05).

In general, all samples at *T*_f_ were correlated with high DOC and TDS. The occurrence of species belonging to the classes of *Alphaproteobacteria, Gammaproteobacteria, Betaproteobacteria, Actinobacteria*, and *Armatimonadia* were positively correlated with higher DOC concentration at *T*_f_. Moreover, the community structure for each treatment at *T*_i_ was clearly separated from those at *T*_f_, except for the soil only treatment (**Figure 4**). Surprisingly, two samples from this treatment were very different from all others at both *T*_i_ and *T*_f_, suggesting that it may be affected by runoff collection or may be have been contaminated during the filling of the incubation tanks.

### Taxonomy of Core Micro-biomes

Of the original 327,327 raw sequences, 17,017 OTUs remained after data processing. Core OTUs were OTUs which abundances constituted >1% of the total OTU abundance in at least one sample. The core community represented in all samples encompassed 136 OTUs but 27% of those could not be classified at the class level.

**FIGURE 5:**
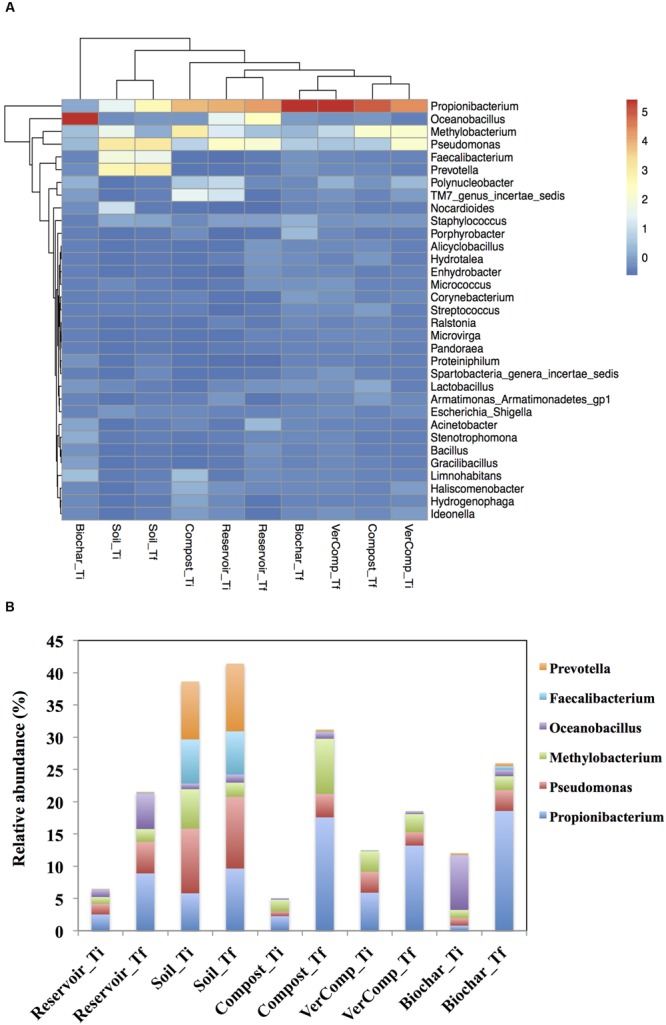
**(A)** A heatmap showing the comparison and cluster analysis of microbial composition in the pool samples. Indicator scores are based on the OTU abundance in the microbial assemblages. Only the OTUs with a frequency of >1% in at least one sample were shown in the figure and **(B)** Comparison of abundance of some genera in all treatments between *T*_i_ and *T*_f_.

Due to the large variation among the replicated mesocosms, the differences in abundance of most phyla were not statistically significant among the treatments. A comparison and cluster analysis of microbial composition in the pool samples was performed to assess dominances at the genus level (**Figure 5A**). At T_i_, *Pseudomonas* spp. (*Proteobacteria*), *Propionibacterium* spp. (*Actinobacteria*) and *Methylobacterium* spp. (*Proteobacteria*), *Faecalibacterium* spp. (*Firmicutes*) and *Prevotella* spp. (*Bacteroidetes*) dominated the communities in the soil-only runoff incubations with the relative abundance of 10, 5.8, 6.11, 6.8, and 8.9%, respectively. *Oceanobacillus* spp. (*Firmicutes*) (8%) were also highly abundant in the biochar runoff incubation whereas *Propionibacterium* spp. were intermediately abundant in the mesocosms that had received compost (2.27%) or vermi-compost (5.9%) runoff, or in the reservoir water controls (2.5%). *Methylobacterium* spp. were also abundant in the mesocosms with both compost and vermi-compost runoff additions with the relative abundance of 1.84 and 3.19%, respectively (*P* < 0.05; **Figure 5A**).

After 16 days of incubation, clear differences could be observed in bacterial community structure. The relative abundance of *Propionibacterium* spp. increased in all samples with the highest relative abundance in the mesocosms with biochar and compost runoff addition (18.6 and 17.6%, respectively) and *Pseudomonas* spp. was found with higher relative abundance in reservoir water control (4.89%) and soil-only runoff incubation (11.2%) compared to mesocosms with organic fertilizers addition at both *T*_i_ and *T*_f_. The relative abundance of *Oceanobacillus* spp. increased sharply in the reservoir water mesocosms by 88.9% but decreased in those that had received the biochar runoff by 77.4%. On the other hand, *Methylobacterium* spp. were present in high abundance in the mesocosms that were impacted with runoff water from the compost treatment (8.55% relative abundance), and they rose in the mesocosms that had received compost or biochar runoff, or in the reservoir controls and decreased in the soil control compared to those at *T*_i_ (*P* < 0.05). The genera of *Faecalibacterium* spp. *and Prevotella* spp. also increased and still dominated the communities in the soil only runoff mesocosms at *T*_f_ compared to other treatments (**Figure 5B**).

## Discussion

We conducted a 16-day mesocosm incubation investigation into the impact of organic fertilizer runoff derived DOC on the structure of an aquatic microbial community. Although less ‘naturalistic’ than *in situ* environments, we show that the addition of DOC from organic amendments can alter the structure of microbial communities in such systems, reducing evenness and richness as compared to a reservoir water control. Performing realistic rain application rates on soil parcels under usual fertilizer application rates of compost, vermi-compost and biochar, we found significantly higher DOC concentrations in runoff relative to that of the soil control. Previous studies have also shown that the application of organic carbon from different sources into soil increased the concentration of DOC in surface runoff compared to DOC runoff from bare soil. For example, Gandolfi et al. (2010) observed increases of DOC concentration of 6–17 times in surface runoff when corn residues were applied into soil. Janeau et al. (2014) also observed lower DOC export rates in bare soils from the Dong Cao catchment area relative to those with mulch or plant cover.

The addition of runoff to reservoir water resulted in some variability at *T*_i_ in both DOC concentrations and in bacterial diversity as a consequence of the different additions. However, in comparison to these allochthonous inputs of DOC at the start of the experiment, the increases in DOC concentration after day 5 were probably from autochthonous sources e.g., primary production as there was a large increase in Chl*a* (up to 72.8 μg L^-1^) over the first few days. This then declined (**Figure 2B**), suggesting the crash of an algal bloom. The excretion of DOC by phytoplankton during photosynthesis is one of the main sources of DOC in aquatic systems (Mague et al., 1980; Biersmith and Benner, 1998). Indeed, given the high concentrations of Chl*a*, it is perhaps not surprising that in some incubations DOC concentration almost doubled. DOC is released during active photosynthesis (exponential growth) but also when phytoplankton biomass becomes stable (stationary phase) or when nutrient limitation and cell death occurs (Biersmith and Benner, 1998; Rochelle-Newall et al., 2008; Rochelle-Newall et al., 2014). This may explain why no direct correlation was observed between Chl*a* and DOC concentration. Similar decoupling of DOC dynamics and Chl*a* concentration has been observed in other systems: Sommaruga et al. (1999) found no significant correlation between DOC concentration and Chl*a* in an Austrian lake. Kim et al. (2000) working in Lake Soyang also reported that the high DOC concentrations observed were due to the presence of cyanobacterial blooms.

### Autochthonous vs. Allochthonous DOC as a Structural Factor for Bacterial Communities

Over the course of our experiment, the richness and the evenness of bacteria in mesocosms receiving runoff water from soil amended with organic fertilizers decreased in parallel to an increase in DOC concentration. The only exception was in the mesocosms that had received compost runoff addition were evenness increased. In contrast, the richness of the bacterial communities in the reservoir water and soil only treatments remained unchanged and evenness of the communities increased between *T*_i_ and *T*_f_ (**Figure 3**). DOC serves as an important substrate and energy source for heterotrophic bacteria (del Giorgio and Davis, 2003; Wei et al., 2015a) and the metabolic activity of the community is controlled by DOC quantity and quality and, hence, carbon source (Pommier et al., 2014). In our experiment, the DOC additions in the reservoir water were principally from phytoplankton production (an autochthonous source), whereas in all the other treatments, DOC inputs were initially from soils (allochthonous) and after 6 days from primary production (autochthonous). Thus the autochthonous vs. allochthonous ratio of carbon source may be an important structural factor influencing bacterial community composition during the incubation. Such results are coherent with those from Attermeyer et al. (2014) who did not observe shifts in bacterial community composition with increasing additions of autochthonous DOC, but did observe a shift when both allochthonous and autochthonous sources were present. Indeed, it has been suggested that bacterial taxa partition along a C source gradient with different ratios of allochthonous vs. autochthonous C (Kritzberg et al., 2006; Jones et al., 2009). Evenness in the reservoir water and soil only treatments also increased, which may represent the stimulation of bacterial species specialized in phytoplankton derived organic matter, which are generally considered as highly bioavailable (Rochelle-Newall et al., 2008, 2011). Interestingly, richness also decreased in the mesocosms with biochar, compost and vermi-compost runoff additions, suggesting a specialization of species when both carbon sources were present. Regarding the biochar addition, some bacteria species have been hypothesized to preferentially degrade more recalcitrant soil OM constituents (Anderson et al., 2011) and this may well explain the decreased richness observed in this addition.

### Changes in Community Structures and Key Taxa Correlated with DOC Decomposition

Community structures were affected by the incubation although this seemed less marked in the soil-only control incubations (**Figure 4**). DOC and pH changes related to these incubations appear to be the main factors that affected community structure. For example, the relative number of sequences assigned as *Actinobacteria* increased with increasing DOC and pH during the course of the experiment. Similarly, the relative number of sequences representing *Alphaproteobacteria, Gammaproteobacteria*, and *Betaproteobacteria* groups were positively correlated with DOC. Previous studies also indicated that pH is an important predictor of bacterial composition and distribution at the continental scale (Lauber et al., 2009). The relative abundance of *Proteobacteria* groups has been shown to be positively correlated to carbon availability (Fierer et al., 2007) and to soil pH and organic C concentrations (Wei et al., 2015a,b). Similarly, Wei et al. (2015a) also showed that *Alphaproteobacteria, Gammaproteobacteria*, and *Betaproteobacteria* groups significantly increased in compost amended soil. The *Actinobacteria* phylum was always predominant across many different finished composts (Franke-Whittle et al., 2009). *Actinobacteria* are considered important during composting process because of the ability of this group to decompose cellulose and chitin. Moreover, they are considered to play a crucial role in the bio-stabilization of refractory organics (Vivas et al., 2009; Federici et al., 2011), the degradation of recalcitrant compounds and, the suppression of pathogenic microorganisms through the secretion of various antibiotic compounds (Franke-Whittle et al., 2009).

Two key genera were found to respond to the different treatments during the incubation. At *T*_f_, there was a significant increase in *Propionibacterium* spp. in all treatments, especially in the biochar runoff incubation where the highest number of sequences was found. *Propionibacterium* spp. are a group of gram-positive bacteria that are generally considered as anaerobic to aerotolerant but that prefer anaerobic conditions for growth. *Propionibacterium* spp. utilize large and complex molecules and convert them into smaller molecules. The production of propionic and acetic acids during lactic acid metabolism is one such example (Wang and Yang, 2013). Interestingly, Gomez et al. (2014) indicated that the addition of biochar in soil shifted the microbial community towards gram-negative bacteria relative to other types of microorganisms, further supporting the suggestion that biochar, although bringing a relatively low addition in terms of DOC, did exert a strong role on bacterial community composition when added to reservoir water. *Methylobacterium* spp. was also dominant in the high DOC concentration compost treatment. These bacteria gram-negative *Alphaproteobacteria* are known to be abundant in compost (Danon et al., 2008) as well as in soils, lakes and sediments (Rekadwad, 2014). The genus *Methylobacterium* is known to utilize methane and other more complex organic compounds (Yim et al., 2014). Propionic acid, which is produced by *Propionibacterium* spp., can be utilized as a sole carbon source by *Methylobacterium* spp. (Gallego et al., 2006) indicating a potential metabolic relationship between these two genus. *Propionibacterium* spp. and *Methylobacterium* spp. may thus represent sentinel species for ecosystem changes, particularly in systems with high inputs of allochthonous organic matter (Salvador Pedro et al., 2001).

## Conclusion

Understanding the off-site impacts of agricultural activities is critical if we are to preserve the ecological integrity of managed ecosystems. Our research indicates significant effects of organic fertilizers on the downstream aquatic microbial community in a tropical system. Here we show that realistic amounts of runoff from amended and non-amended soils can provide a large source of allochthonous DOC. In addition, that this allochthonous DOC has a strong impact on microbial community structure and composition in an experimental aquatic system. We found a reduction in both the richness and evenness of the communities with added allochthonous organic matter which may point towards a specialization of the communities, similar to what was previous observed in a short term (72 h) incubations (Pommier et al., 2014). Moreover, the data suggest that DOC and pH are the main factors controlling community structure in mesocosm experiments. Ultimately, we identified two sentinel genera, i.e., *Propionibacterium* spp. and *Methylobacterium* spp., which could be important to track using e.g., targeted quantitative PCR, to monitor ecological changes in sites under high OM inputs. These results further underline that even though organic composts can be considered as being less damaging for the environment as compared to other chemical additions, the full consequences of their application need to be taken into account, especially in agricultural practices of tropical countries.

## Author Contributions

DT, ER-N, JJ, ML, CH, TP designed the experiments; CH, QT, DT, ML, HT, DO, JJ, AM, and ER-N carried out the work; HL, CH, TP, ER-N interpreted the results and HL, TP, and ER-N wrote the manuscript that was revised and improved by all the coauthors.

## Conflict of Interest Statement

The authors declare that the research was conducted in the absence of any commercial or financial relationships that could be construed as a potential conflict of interest.
